# Studbook and molecular analyses for the endangered black-lion-tamarin; an integrative approach for assessing genetic diversity and driving management in captivity

**DOI:** 10.1038/s41598-020-63542-2

**Published:** 2020-04-22

**Authors:** Paola Andrea Ayala-Burbano, Pedro Manoel Galetti Junior, Dominic Wormell, Alcides Pissinatti, Mara Cristina Marques, Patrícia Domingues de Freitas

**Affiliations:** 1 Department of Genetic and Evolution, Federal University of São Carlos, São Carlos, SP Brazil; 2 Durrell Wildlife Conservation Trust, Trinity, Jersey England; 3 Rio de Janeiro Primatology Center, Rio de Janeiro, RJ Brazil; 4 Zoological Park Foundation of São Paulo State, São Paulo, SP Brazil

**Keywords:** Animal breeding, Inbreeding

## Abstract

Breeding strategies based on molecular markers have been adopted by *ex-situ* conservation programs to assess alternative parameters for the genetic diversity estimates. In this work we evaluated molecular and studbook data for captive populations of black-lion-tamarin (BLT), an endangered primate endemic to Brazil’s Atlantic Forest. Pedigree analyses were performed using BLT studbook information collected from 1973 to 2018. We analyzed the whole captive population since its foundation; the current captive population (CCP); and all extant BLTs in the Brazilian captive population (BCP), separately. Microsatellite analyses were implemented on the BCP individuals from the eighth generation (BCP-F8) only to avoid generation overlap. The expected heterozygosity for BCP-F8, using molecular, data was 0.45, and the initial expected heterozygosity was 0.69. Kinship parameters showed high genetic relationships in both pedigree and molecular analyses. The genealogy-based endogamy evidenced a high inbreeding coefficient, while the molecular analyses suggested a non-inbreeding signature. The Mate Suitability Index showed detrimental values for the majority of potential pairs in the CCP. Nevertheless, some individuals evidenced high individual heterozygosity and allele representation, demonstrating good potential to be used as breeders. Thus, we propose the use of molecular data as a complementary parameter to evaluate mating-pairs and to aid management decision-making.

## Introduction

Captive breeding programs have been recognized as a powerful alternative for rescuing endangered species and for biological conservation^1,2^. Often based on pedigree analyses, *ex-situ* management plans aim to maintain demographically stable populations, retaining genetic diversity, limiting inbreeding, and avoiding adaptation to captivity^1,3–6^. However, this is not an easy task, and consequently captive groups tend to present lower levels of genetic diversity and higher inbreeding rates than expected^2,7^, challenging the success of these captive breeding programs. On the other hand, wild endangered species often present small and fragmented populations subjected to bottleneck effects and absence of gene flow, and low genetic diversity levels are commonly also observed in nature^8,9^. This is the case for the black-lion-tamarin (BLT), *Leontopithecus chrysopygus* (Callitrichidae, Platyrrhini), an endangered primate inhabiting exclusively the Atlantic Forest of São Paulo state in Southeast Brazil^8,10^.

The population size of *L. chrysopygus* in nature is small^11^, currently estimated at a total of a thousand individuals living in a few small forest fragments^12^. This species was assumed to be extinct about 65 years, when a small population was rediscovered in the Morro do Diabo State Park (SP, Brazil)^13^. At that time, a population census estimated that only about 200 animals existed in nature. In 1973, the first seven wild individuals of two contiguous subgroups of BLT were brought into captivity, at the Biological Bank of Tijuca in Rio de Janeiro (Rio de Janeiro, Brazil)^14^. In 1985, because of the construction of the Rosana Hydroelectric dam, invading about 3,000 ha of the protected Morro do Diabo State Park, eight wild groups were rescued. Of these animals, one group of six individuals was brought to the Rio de Janeiro Primatology Center (CPRJ; Guapimirim, RJ, Brazil), and the seven other groups, totaling 31 BLTs, were kept in a vivarium, and transferred to a nearby forest fragment later^15,16^. However, due to the poor health condition of these transferred animals, only sixteen BLTs (six males, eight females and two animals with no gender information) survived and were relocated to the Zoological Park Foundation of São Paulo State (FPZSP; São Paulo, SP, Brazil), starting a new group in captivity in 1986^17^.

In 1987, the International Committee for the Preservation and Management of BLTs was organized in order to contribute to the management of the captive groups of this species. From this initiative, the studbook for the black-lion-tamarin, describing genealogical records for the captive animals, was created in the same year^18^. The first captive group of BLT overseas emerged in 1990, when six individuals were transferred from CPRJ to the Jersey Wildlife Preservation Trust (Jersey Zoo, Jersey, Channel Islands), currently known as Durrell Wildlife Conservation Trust (DWCT). The animals kept at Jersey Zoo successfully produced offspring, and some *L. chrysopygus* were transferred to other institutions in Europe, North America and Australia. However, the majority of these individuals died^17^, and nowadays there are only extant captive BLT overseas in Jersey^8^.

Similarly to most *ex-situ* breeding programs, the management of *L. chrysopygus* in captivity has been implemented based only on pedigree analyses^8^, aiming to minimize population average kinship and preserve representative genetic diversity^19–21^. Although this strategy has been considered appropriate to avoid inbreeding^22^, even if a pedigree has been properly scored for a captive group since its foundation, founder relationships are generally unknown, and for management purposes it is commonly assumed that the founders are unrelated^23^. Moreover, captive breeding programs often recruit few founders, in general from a single population, representing a small proportion of the total genetic diversity of a species^1^.

To compensate for the lack of knowledge about the initial genetic diversity and relationships between the founders, various institutions that manage endangered species have recently tried to combine molecular data with pedigree analyses^6,24–28^, although studies integrating both types of data are still scarce^29^.

In the present work, we performed studbook and microsatellite analyses to assess population genetic structure and infer demographic and genetic diversity parameters in the captive groups of *L. chrysopygus*. We analyzed molecular and pedigree data and estimated genetic diversity for F0. The most common pedigree-based index used to choose mates in breeding programs was compared with the individual heterozygosity obtained by microsatellite markers. Our findings suggest that although genealogical analysis has been beneficial, an integrated approach including molecular data might be useful for a better understanding of genetic diversity and the structure of the BLT population in captivity, and for proper metapopulation management.

## Results

### Genealogical and demographic inferences based on pedigree data analyses

The whole captive population of BLTs consists of 517 animals (Supplementary Fig. S1), of which 466 have already died, including 35 wild founders and three individuals with unknown parents (Table 1). The Brazilian captive population of BLTs includes 37 adults recorded in the 2014 studbook^30^. Of these, 17 individuals were maintained at the Primatology Center of Rio de Janeiro, 16 at the Zoological Park Foundation of São Paulo, and four at the São Carlos Ecological Park (PESC; São Carlos, SP). However, some of these animals were relocated among the zoos during the years 2015–2018, including five BLTs that were recently transferred from both CPRJ (two animals) and FPZSP (three animals) to Jersey Zoo. In addition, other BLTs were born and one wild individual from Patrania municipality was brought to captivity. Currently, there are 55 living animals in the captive population. Eight of them are in Jersey Zoo, one in Magdeburg Zoo (German) and 46 are in Brazil (15 at CPRJ, 26 at the FPZSP, three at PESC, and two at Belo Horizonte Zoological, (BH Zoo; in Minas Gerais state) (Supplementary Table S1).

**Table 1:** Founders’ data registered from 1973 to 2018 in the International Studbook for the black-lion-tamarin (BLT), showing local of capture, transfer location, year of capture, number of individuals captured, and the current status of the fragments where BLTs still occur.

Founders introduced from nature				
Local of capture	Transfer location	Year of capture	Number of individuals	Fragments with BLTs
Morro do Diabo State Park	CPRJ	1973	7	yes
Morro do Diabo State Park	CPRJ	1985	4	yes
Morro do Diabo State Park	FPZSP	1986	14	yes
Morro do Diabo State Park	FPZSP	1987	1	yes
Ribeirão Bonito Farm	FPZSP	1991	3	no
Wild (Missing location)	PEMQB*	1998	1	—
Wild (Missing location)	CPRJ	1999	1	—
Buri	Sorocaba	2003	1	no
Buri	FPZSP	2007	1	yes
Morro do Diabo State Park	FPZSP	2014	1	yes
Pratania	FPZSP	2017	1	yes

Zoological Park Foundation of São Paulo (FPZSP), Primatology Center of Rio de Janeiro (CPRJ), *Municipal Zoo Park Quinzinho de Barros (PEMQB*). - Missing information.

**Figure 1:**
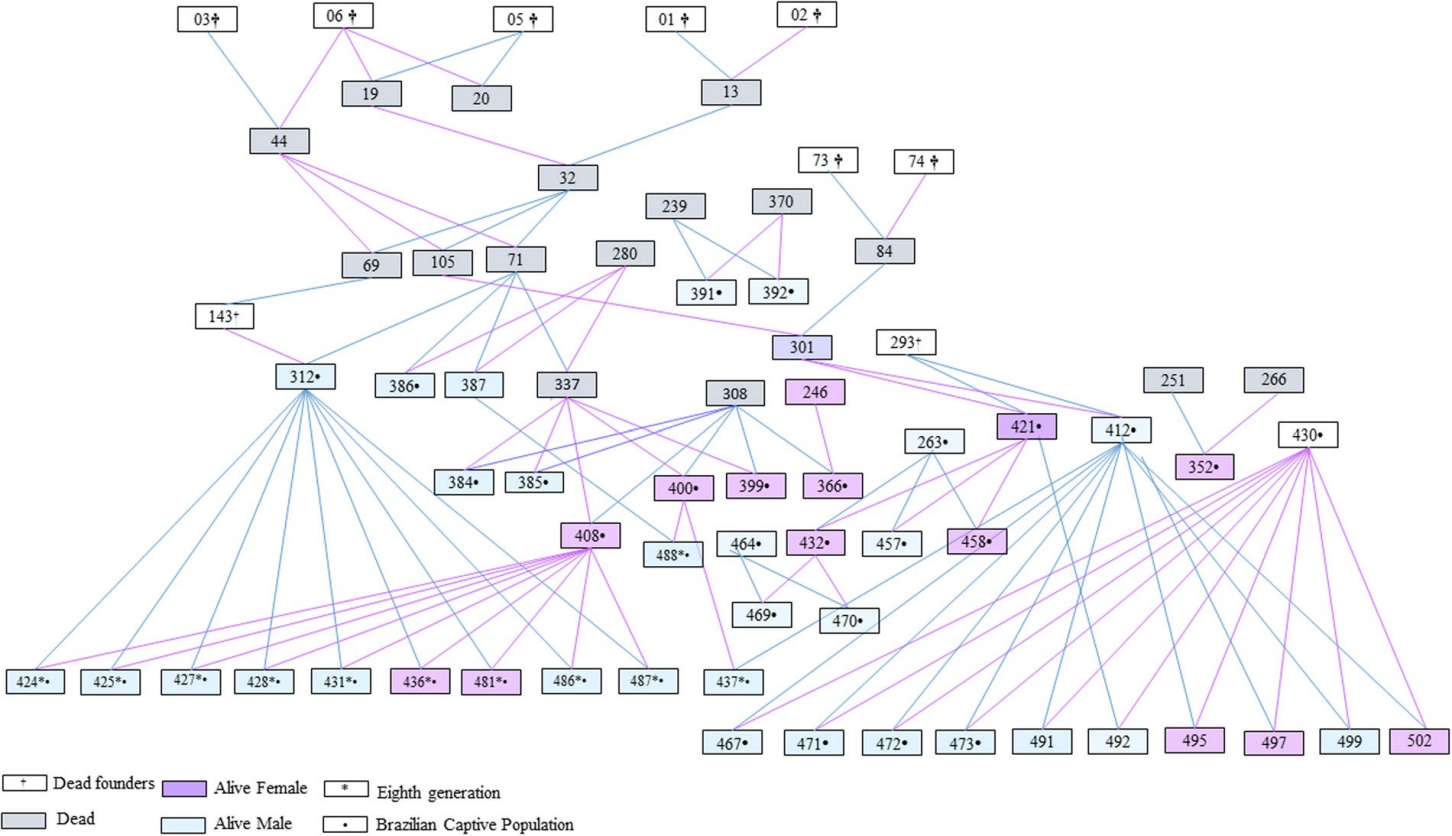
Kinship tree depicting the 37 black-lion-tamarins in the Brazilian Captive Population (BCP) and the 11 individuals in the eighth generation (BCP-F8). All BCP individuals descend from 10 wild animals, of which only one (430) is still living. Note the current severe skew in breeding contribution. White squares: founders; grey squares: dead individuals; green squares: alive males; and purple squares: alive females.

The pedigree graphical representation for the WCP revealed nine generations of BLTs in captivity up to 2018, with several non-breeding individuals and some others showing higher reproductive rates (Supplementary Fig. S1). Related to the pedigree depth, until the fifth generation back, the completeness level for the WCP was 92% for the parent generation, 71% for the grandparent generation and 45% for the great-grandparent generation. The Brazilian captive population showed an overlap of generations including animals from the 5th, 6^th^, 7^th^, and 8^th^ generations. Thus, for the integrative approach, we calculated genetic diversity estimators for the individuals comprising the eighth generation (BCP-F8), which included the descendants from the prior generations without the parents (Fig. 1).

The longest generation interval values were found for father-daughter for the WCP (6.32 years) and father-son for the CCP (11.04 years) populations. The average generation interval for WCP and CCP was 5.44 and 7.57 years, respectively. The generation intervals calculated across all pathways are presented in Table 2. More details of demographic and genealogical results, including age structure, fertility (*Mx*), mortality (*Qx*), survival *(Lx*), expected lambda-λ, instantaneous rate of change of the population (*r*), and reproductive peaks in captivity are shown in Supplementary Information (Figs. S2–S6).

**Table 2:** Mean of generation interval (in years), considering the four paths (father-son; father-daughter, mother-son and mother-daughter) in the whole (WCP) and the current captive (CCP) populations of black-lion-tamarin. Number of individuals (N), Standard deviation (SE).

Parents-offspring	WCP			CCP		
	N	Years	SE	N	Years	SE
Father-Son	53	5.76	3.35	7	11.04	4.24
Father-Daughter	58	6.32	2.68	5	8.81	3.36
Mother-Son	54	4.33	2.25	7	5.24	1.76
Mother-Daughter	58	5.29	2.29	5	4.71	1.87
Total	223	5.44	2.75	24	7.57	3.94

### Measures of the probabilities of gene origin based on pedigree data

The effective number of ancestors (*fa*) calculated following Boichard *et al.*^31^ was 10 for WCP and eight for both CCP and BCP. The effective number of founders (*fe*) was equal to 10 for WCP, CCP and BCP (Table 3). These results show a markedly lower number of non-captive potentially contributing individuals than the total number of wild animals registered in the current version of the BLT Studbook (Table 1). For CCP, the proportion of remaining genetic diversity (*rGD*) based on the founder genome equivalent (*fge*) was 87.2%. When we considered only BCP, *rGD* was equal to 83.9% (Table 3).

**Table 3:** Demographic and gene origin statistics for the whole (WCP), current (CCP) and Brazilian captive (BCP) populations of black-lion-tamarin.

	WCP	CCP	BCP
Number of individuals	517	55	37
Number of founders	35	13	13
Effective number of founders (*fe*)	10	10	10
Effective number of ancestors (*fa*)	10	8	8
Founder genome equivalent (*fge*)	27	3.93	3.12
Remaining genetic diversity (*rGD*) (%)	—	87.2	83.9

### Inbreeding, mean kinship and effective population size based on pedigree data analyses

**Figure 2:**
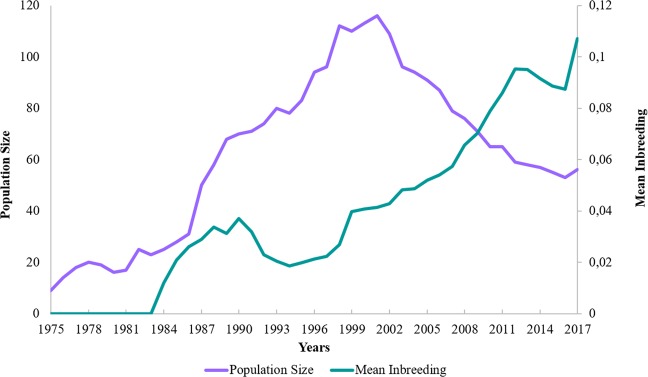
Variation in mean inbreeding and population size in the whole captive population of black-lion-tamarin (BLT/WCP).

The inbreeding coefficient (*F*) was higher in BCP and CCP than in WCP (Table 4). The inbreeding values ranged from 0.0119 in 1984 to 0.1070 in 2018 (Supplementary Table S2), showing a curve fluctuating according to the number of inbred individuals in each year (Fig. 2).

**Table 4:** Inbreeding statistics (*F*) for the whole (WCP), current (CCP) and Brazilian (BCP) captive populations of black-lion-tamarin. Unk (Unknown).

	WCP				CCP				BCP		
	Total	Male	Female	Unk Sex	Total	Male	Female	Unk Sex	Total	Male	Female
N° of records	517	242	188	87	55	32	15	8	37	24	13
*F*	0.052	0.111	0.134	0.338	0.101	0.169	0.234	0.226	0.108	0.092	0.112
Minimum F	0.000	0.000	0.000	0.000	0.000	0.000	0.000	0.000	0.000	0.000	0.000
Maximum F	0.338	0.338	0.226	0.226	0.395	0.395	0.216	0.395	0.225	0.225	0.215

Mean kinship statistics showed slightly superior values in the CCP and BCP (Table 5). The realized effective population size (*NeI*) and the value of *Nec*, which assumes random mating occurring in the near future, are shown in Table 5. The ratio *Nec*/*Ne* for CCP was equal to 0.30.

**Table 5:** Effective population size and mean kinship for the whole (WCP), current (CCP) and Brazilian (BCP) captive populations of black-lion-tamarin.

	WCP	CCP	BCP
Effective population size (*NeI*)	—	16.85	15.35
Effective population size (*Nec*)	—	11.58	12.59
Mean equivalent generation (*ge*)	2.64	3.66	2.61
Mean kinship (*Mk*, %)	13.34	12.74	16.04

The genetic structure, based on Wright’s *F*-statistics (*F*_*ST*_ between groups) and mean coancestry (*fij*) within and between zoos, is shown in Table 6. The *F*-statistics evidenced that Jersey Zoo and CPRJ are the most genetically distant captive groups, whereas mean coancestry and *F*_*ST*_ values showed that PESC and CPRJ are the most related ones.

**Table 6:** Genetic structure based on Wright’s *F-statistics*. *F*_*ST*_ values below the diagonal, and mean kinship between zoological parks (*Mk*) above the diagonal. Mean coancestry (*fij*), within (diagonal) and between subpopulations (off diagonals), for the current population (CCP) of black-lion-tamarins.

*F*_*ST*_ *– Mk*					Mean Coancestry (*fij*)				
Zoos	Jersey-Mag	CPRJ	FPZSP	PESC	Zoos	Jersey	CPRJ	FPZSP	PESC
Jersey-Mag		0.136	0.095	0.073	Jersey-Mag	16	12	9	8
CPRJ	0.106		0.070	0.139	CPRJ	12	31	8	13
FPZSP-BH	0.022	0.077		0.055	FPZSP-BH	9	8	12	6
PESC	0.044	0.008	0.021		PESC	7	13	6	23

### Genetic diversity inferences based on molecular and pedigree data integrative analyses

For the integrative approach, we considered the eleven individuals from BCP in the eighth generation (BCP-F8), which were born between 2005 and 2012. Currently, 10 of these are alive and consequently are included in the CCP as well (Fig. 1). The set of 15 microsatellite loci was successfully amplified in these samples, with no indication of null alleles, stuttering, allelic dropout and significant LD (p > 0.05). Despite literature report some issues for dinucleotide loci^32–34^, no genetic inconsistencies were found for these loci, after following the technical procedures employed for DNA amplification and genotyping (see Supplementary Information). The obtained electropherograms (EPGs) evidenced specific allele patterns with proper quality (Supplementary Fig. S8). After sequencing and alignment of the amplicons, the expected motifs were searched, and the microsatellite sequences were confirmed for all heterologous loci.

In total, we computed 31 alleles, ranging from two to three per locus, with an average of 2.06 alleles per locus, and average allelic richness equal to 2.07. The equivalent genetic diversity estimators, based on both pedigree and molecular data, are shown in Table 7. The effective population size (NeI) showed a higher value when calculated through pedigree analyses. The ratio of Ne/N was equal to 0.18 and 0.12 for genealogical and molecular data, respectively. The founder genome equivalent was 1.42, and the mean effective number of alleles was 1.84. The degree of kinship (*Mk*) based on pedigree data showed a high value concordant with that observed using the pedigree inbreeding index (*F* = 0.19). Kinship based on molecular data (r_m_) confirmed a high degree of relatedness. The molecular inbreeding coefficient was negative (f = −0.58), as a consequence of an excess of heterozygosis, the observed mean (Ho = 0.73) being higher than the expected heterozygosity (He = 0.45).

**Table 7:** Pedigree and molecular genetic diversity indices for the black-lion-tamarins of the Brazilian captive population in the eight generation (BCP-F8).

Pedigree data	*N*	*Ne*	*Ne/N*	*Mk*	*F*	*fge*	*rGD*
BCP-F8	11	2.0	0.18	0.35	0.20	1.42	65%
**Molecular data**	**N**	**Ne**	**Ne/N**	**rm**	**f**	**Nae**	**rGD**
BCP-F8	11	1.4	0,12	0.38	−0.58	1.84	65%

N: Number of individuals; Ne: Effective population size; Ne/N: Relation between effective population size and number of individuals; Mk: mean kinship for pedigree data; rm: average kinship coefficient calculated by molecular markers; F: average pedigree inbreeding; f: average system-of mating inbreeding; fge: founder genome equivalent; Nae: mean effective alleles; rGD: remaining genetic diversity.

**Figure 3:**
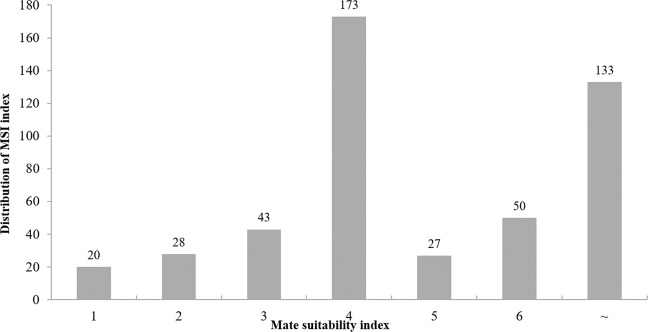
Distribution of the mate suitability indices (*MSI*) for the 55 black-lion-tamarin pairings (480 simulations in total). Scores (1 to ~) indicating 20 mating pairs very beneficial (1); 28 moderately beneficial (2); 43 slightly beneficial (3); 173 slightly detrimental (4); 27 detrimental (5); 50 very detrimental (6); and 133 very highly detrimental (~). Values of IR ranging from -1 to +1. When the IR values are higher, the individual heterozygosity estimates are lower.

The remaining genetic diversity measured by pedigree analyses was 65%. Remaining genetic diversity and heterozygosity for F0, calculated based on the integrative approach, were 65% and 0.69, respectively. The values of *MSI* estimated by PMx for all extant BLTs from CCP, including individuals from Jersey, resulted in a total of 480 simulated potential couples, in which 80% were considered at least as slightly detrimental (4, 5, 6, ~) (Fig. 3). In contrast, the individual heterozygosity, based on the IR Index, for the three individuals from FPZP (studbook numbers 471, 472, 497) and the two individuals from CPRJ (studbook number 436, 487), which were recently transferred from Brazil to Jersey, ranged from −0.654 to −0.088 (Table 8), indicating high heterozygosity.

**Table 8:** Information for the black-lion-tamarins transferred from the Zoological Park Foundation of São Paulo (FPZSP) and Primatology Center of Rio de Janeiro (CPRJ) in Brazil to Jersey Zoo.

Studbook number	Sex	Age (years)	Origen	Generation	Internal relatedness (IR)
436	Female	8	CPRJ	8	−0.410
487	Female	5	CPRJ	8	−0,589
497	Female	4	FPZSP	6	−0.590
471	Male	5	FPZSP	6	−0,088
472	Male	5	FPZSP	6	−0.654

## Discussion

Setting up an efficient captive breeding program requires a precise knowledge of the genetic diversity and genealogical data of the populations to be managed^35^. In this sense, pedigree analyses can provide relevant information for the management of species in captivity^28^. However, the effectiveness of pedigree-based approaches depends on its completeness and depth, since deeper pedigrees usually generate more accurate and robust inferences^35^. According to the present study, 92% of ancestral relationships in the whole captive population of BLTs are well known. Taking into account such high pedigree depth value, the demographic and genetic inferences raised herein, based on the BLT studbook data, should be considered as reliable.

Overall, the captive BLT population is well established; nevertheless, its age structure is typical for a slowly growing population (λ > 1), showing an explicit decline from the year 2001, in both sexes. The whole captive group reached a maximum population size of 114 (59 males and 55 females) in 2000, but in the subsequent years it suffered a drastic and continuous decline, though new births have contributed to the growth of the current captive population in Brazil. We also observed an increase in the average generation interval over the years. These results are quite probably due to management efforts aimed at minimizing inbreeding by the reuse of less related animals as reproducers^36^, that in this case are the oldest BLTs. In addition, we verified low rates of reproduction of founder and non-founder wild animals, and also a high number of unrelated captive animals that never reproduced at all. Thus, despite the huge efforts to avoid matings between closer relatives, or in recent years to decrease accumulation of inbreeding as much as possible, the BLT captive population has been showing an increase in inbreeding over time and high kinship values.

High rates of inbreeding and kinship can promote genetic diversity loss and inbreeding depression in future generations, compromising fitness-related features such as viability, birth weight and fecundity^37^. Unfortunately, some possible evidence of inbreeding depression, such as bone deformities, low copulation rates, low sperm motility, high rates of infertility and cleft lip that cause infant deaths during tooth changes, have already been observed in the Brazilian captive population of BLTs (Pissinatti, A., personal communication). Additionally, the European population has experienced a high incidence of gallbladder problems (Wormell, D., personal communication). In spite of this, the short-term goal of the BLT breeding program in captivity has been achieved by the high survival rates in the infant and juvenile age groups. However, the long-term goal for BLT management consists of maintaining genetic diversity levels and avoiding inbreeding depression^38^.

Captive populations often have a very small number of founders, which are considered unrelated and consequently have inbreeding rates assumed as zero^1,23^. In our study, we know the origin of the founders, which came from two neighboring groups of the same population that lived in the Morro do Diabo State Park and might be genetically related. Consequently, the kinship and inbreeding values calculated by pedigree analyses were high, compromising the viability^38^ of the captive population of BLTs in the long-term.

Fortunately, according to measures of probabilities of gene origin, our data showed a greater value of effective size when compared to the effective number of founders, ancestors and founder genome equivalent values. The relationship between the effective size and the population size (NeI/N) shows that the pedigree-based recommendations are being directed to the equalization of the families, and the sex ratio and the number of individuals throughout the generations, aspects considered very advantageous^39^. Moreover, molecular data have shown a higher observed heterozygosity than expected, which leads us to infer that zoos are managing the population appropriately. Nevertheless, the PMx analyses showed that the *MSI* values are at least detrimental^40^ for the majority of potential couples of the CCP. From the total of simulated mate pairs, 174 and 30 showed values of *MSI* that were slightly detrimental and detrimental, respectively. The remaining ones were considered very detrimental and very highly detrimental. On the other hand, only 20% pairs had *MSI* values considered as beneficial for breeding programs, whereas the BLTs recently transferred from Brazil to Jersey showed *MSI* values moderately or slightly beneficial when we simulated pairing with BLTs from Jersey Zoo.

The *MSI* is the most common parameter used to select mates in captive breeding programs, and considers differences in genetic diversity, kinship, inbreeding coefficient and unknown ancestry, all calculated only by pedigree data^40–42^. Considering the *MSI* values found here, we estimated the Internal Relatedness index as a complementary parameter to the *MSI*, in order to gain some insights based on molecular data as well.

The IR index is a method for estimating individual heterozygosity and considers that rare alleles count more than common alleles. Negative IR values indicate higher heterozygosity, whilst positive values are attributed to more homozygous individuals^43^. In our study, despite the fact that the transferred BLTs have shown moderately or slightly beneficial *MSI* values, they all showed negative IR values, indicating that these individuals have high heterozygosity, besides allele representativeness, and consequently are valuable as breeders^44^. In fact, these animals have already mated and successfully produced offspring in Jersey (Wormell, D., personal communication; https://www.durrell.org/wildlife/news/durrell-celebrates-birth-endangered-monkeys/). Alternatively, if only very homozygous individuals are available for forming mate-pairs, genetic differentiation among the potential breeders and their allele representativeness^45^ must be considered in addition to *MSI* scores.

Management decisions must take into account the possibility of changes in genetic diversity by mating between genetically more divergent individuals^1,45^. Previous molecular analysis, using the same set of microsatellites used here, showed private alleles in each captive group from Brazil and Europe, evidencing genetic structuring among them^8^. In addition, the pedigree analyses performed here pointed to greater genetic differentiation between the Jersey and CPRJ captive groups. It is noteworthy that the animals that successfully mated in Jersey are from CPRJ (487) and FPZSP (472), these latter being descended from the CPRJ group. In this case, the genetic diversity increment was beneficial to the metapopulation management of BLTs^46^.

Notwithstanding this, changes in the genetic diversity of source and recipient populations, by movement of individuals, are not always beneficial to both populations. Such groups need to be carefully managed to maintain the maximum of allele richness, to avoid inbreeding, but also potential outbreeding depression. Therefore, combining multiple genetic diversity measures, based on both molecular and studbook data, might produce a more robust data set^42,47–49^.

When the initial genetic diversity of the founding captive population is unknown, it is hypothetically considered equal to 1, as proposed by the PMx model commonly used for calculating pedigree parameters^5,40^. However, our findings suggest that despite the remaining genetic diversity is about 65% in both pedigree and integrated analyses, the expected heterozygosity represented in the founder individuals, based on the integrative approach, would be about 0.69. Such results show a more coherent value of genetic diversity for F0, reinforcing the idea that genetic diversity inferences must be specific for each breeding program and cannot be extrapolated from hypothetical assumptions^7,48^.

Molecular analyses are essential for populations with unknown genetic diversity and can be relevant to monitoring genetic diversity across generations in conservation actions^50–53^. According to recommendations of the ISFG (International Society for Forensic Genetics) for the area of non-human DNA typing^34^, they have also potential to be used, by the community of forensic scientist, for investigations involving poaching, smuggling and illegal trade of protected species^32,54,55^

DNA-based studies can still simulate, estimate and compare genetic diversity levels in breeding programs^56^. Genetic management of threatened species has experienced an increase in the last few years^6,29,57,58^, and more recently has been improved by a combination of pedigree and molecular information^59^. For the BLT captive breeding program, we highlight that an integrative approach could be of benefit in terms of allele representativeness and also for considering a more plausible genetic diversity estimate for the founding population.

Overall, to promote the long-term success of the BLT conservation program, we recommend including genetic diversity parameters based on molecular data, in addition to the pedigree analyses and *MSI* scores. Microsatellite-based values of expected heterozygosity, individual heterozygosity, allele richness, private alleles, population structure, inbreeding and kinship could be monitored over generations, helping to evaluate gains and losses of genetic diversity more effectively, and identifying individuals potentially better suited for reproduction and for relocation in captivity^29,42,47^. Finally, we must take into account that an integrated *in situ* and *ex situ* approach is strongly indicated for the metapopulation management of BLTs and to help shield this species from its imminent risk of extinction, since in nature *L. chrysopygus* has a small population size and a very low genetic diversity level.

## Methods

### Ethical requirements and research permits

The present study was approved by the Ethics Committee on Animal Experimentation (Federal University of São Carlos, São Carlos, São Paulo, Brazil), under CEUA-UFSCAR number 9805200815; the Authorization System and Biodiversity Information of the Chico Mendes Institute for Biodiversity Conservation (Ministry of Environment, Federal Government, Brazil), under SISBIO-ICMBio numbers 50616-1; and the National System of Genetic Patrimony Management and Associated Traditional Knowledge (Ministry of Environment, Federal Government, Brazil), under SISGEN number A411359. The approved experimental protocols included the capture of live animals in captivity, and the anesthesia using direct inhalation equipment and blood collection procedures. The animals were handled by a veterinarian who released them safely after blood collection. These procedures followed all ethical and legal recommendations proposed by the institutional and licensing committee and the American Society of Primatologists for the Ethical Treatment of Non-Human Primates (https://www.asp.org/society/resolutions/EthicalTreatmentOfNonHumanPrimates.cfm).

### Studbook data and pedigree analyses

We analyzed all records of *L. chrysopygus* registered in the International Studbook for the black-lion-tamarin (unpublished current version). We considered all BLTs kept in captivity from 1973 to 2018, including the founders, ancestors and their offspring, and we carried out analyses separately for three set of individuals: the whole captive population (WCP), including all living or dead captive BLTs; the current captive population (CCP), comprising all extant captive individuals maintained in Brazil and overseas until 2018; and the Brazilian captive population (BCP), including only living captive BLT adults in Brazil^8^.

For demographic inferences we implemented three different analyses for evaluating the consequences of the applied random mating system and its evolution over time by using the Endog 4.8 software^60^. First, we calculated the pedigree depth by considering the proportion of known ancestors per generation for each offspring, and then we added the interval of generations, defined as the mean age of parents when their progeny is selected to be parent, considering the relationships between mother-daughter, mother-son, father-daughter and father-son^61^. Finally, we estimated the equivalent complete generations based on the proportions of individuals with both known parents. This parameter is also known as the mean equivalent generation (*ge*) and it is calculated as the sum of all known ancestors $(\frac{{1}^{n}}{2})$, where *n* is the number of the ${i}^{th}$ generation separating an individual from each known ancestor (e.g. parents = 1, grandparents = 2, great-grandparents = 3, …)^62^. The complete pedigree was constructed using Pedigree Viewer version 6.5.2.0^63^.

Fertility (*Mx*) was calculated considering the individual fertility or reproductive potential information for each age class. Mortality (*Qx*) was estimated as the proportion of individuals entering an age class versus animals that died before reaching the age class *x* + 1. Survival (*Lx*) was determined as the proportion of individuals surviving from birth to the beginning of the age group *x*. The proportional change in population size from one year to the next, based on life table calculations (expected lambda-λ), and the instantaneous rate of change of the population, averaged for males and females (*r*), were also estimated. A lambda value greater than one indicates an increase in the population. A value of *r* greater than one also means that the population is increasing. All these estimators were calculated using PMx software^40^.

For the pedigree-based genetic inferences, we determined genetic diversity by calculating the total effective number of founders (*fe*)^64^ and total effective number of ancestors (*fa*)^31^, using the Endog 4.8 software^60^, and founder genome equivalents (*fge*)^64^ using PMx^40^. The degree of remaining genetic diversity (i.e., expected heterozygosity originated by limited numbers of founders and its balanced contribution) was calculated based on the following expression: $\frac{He}{H0}=1-\left(\frac{1}{2fge}\right)$, in which *H0* = 1. The inbreeding coefficient (*F*) was estimated to illustrate the trend in mean inbreeding across years. Likewise, mean kinship (*Mk*) was also calculated as complementary information to that provided by the inbreeding coefficient (*F*). *F*_*ST*_ and mean coancestry (*fij*) were calculated following Caballero and Toro^65,66^, considering the genetic divergence between each pair of zoos which hold the species based on the pedigree data. These latter parameters were calculated using PMx software^40^.

The effective population size (*Ne*) was estimated based on two approaches (*NeI* and *Nec*) implemented in Endog version 4.8^60^. First, *Ne* was calculated to estimate the founder population size and to detect the existence of bottlenecks and possible consequences of the mating strategy, via the individual increase in inbreeding (*NeI*), as proposed by De la Rosa *et al*.^67^. To calculate *NeI*, the coefficient of individual increases in inbreeding (∆*Fi*), determined according to Falconer and Mackay^68^ and modified by Gonzales-Recio *et al*.^69^ and Gutiérrez *et al*.^70^, was used. The modified method proposed by Gutiérrez *et al*.^70^ is considered the most appropriate to analyze permanently subdivided populations. *Ne* was also calculated using the increase in coancestry (*Nec*) proposed by Cervantes *et al*.^71^, which is suitable when mixing of populations becomes a usual practice. We also calculated the ratio of the effective population size (*NeI*) to the census size of living captive-born individuals (*NeI*/*N*). Mate Suitability Index (*MSI*) was determined for all potential pairs in the current captive population of BLTs using PMx^40^.

### Biological samples and molecular analyses

Biological samples of all BLTs from the Brazilian captive population were obtained by collecting about 0.5 mL of fresh blood from each individual, using *vacutainers* containing EDTA (3.6 mg). The animals were anesthetized by direct induction using inhalation equipment calibrated with isoflurane (2–5%) and oxygen (2 L/min), and were then released back into their respective enclosures. Blood samples were stored at −20 °C for subsequent DNA extraction. Genomic DNA was obtained following the phenol protocol^72^. The DNA integrity was confirmed using 1% agarose gels under constant voltage (100 V for 45 min) (Supplementary Fig. S7A) and the quantification was performed using GE NanoVue Plus, GE Healdthcare Spectophotometer.

Polymerase chain reactions (PCRs) for the microsatellite amplifications followed procedures proposed by Ayala-Burbano *et al*.^8^. We firstly tested a panel of 22 loci previously described for *Leontopithecus* species^73–75^ (Supplementary Table S3), and posteriorly selected 15 polymorphic loci. PCR-amplified products were visualized in 2% agarose gel (Supplementary Fig. S7B). Genotyping were performed in an ABI3730XL automatic sequencer (Applied Biosystems, Foster City, CA, USA), using GS 500 Liz size standard, and the alleles were scored in the software Geneious version 6.0.6 (https://www.geneious.com). Each sample genotyped as homozygous was confirmed by a minimum of three replications. We also performed multiple PCRs for random samples, in order to identify genetic inconsistencies, according to recommendations proposed by the ISFG (International Society for Forensic Genetics) for the area of non-human DNA typing^34^. More details related to the technical procedures employed for DNA amplification and genotyping of the STR (Short Tandem Repeats) loci are available in Supplementary Information.

Before the statistical analyses, we estimated the occurrence of null alleles, allelic dropout and stuttering for all scored alleles using Micro-Checker^76^. Subsequently, lack of linkage disequilibrium (LD) between loci was verified in Genepop version 4.0.10^77^. We used the linkage disequilibrium method to assess the effective population size. Genetic diversity parameters were inferred by calculating the number of alleles (Na), effective number of alleles (Nae), expected (He) and observed (Ho) heterozygosity using GenAlEx version 6.4^78^.

The proportion of remaining genetic diversity represented in the eighth generation of the BCP was calculated as $\frac{{\rm{He}}}{{\rm{H}}0}={\left(1,-,\frac{1}{2{\rm{Ne}}}\right)}^{{\rm{t}}}$, in which H0 is the initial heterozygosity in the F0 generation, He is the expected heterozygosity calculated by molecular data, t is the number of generations, and Ne is the number of individuals that produced offspring in a specific generation. Ne was calculated by harmonic mean^79^, where ${\rm{Ne}}=\frac{1}{\frac{1}{{\rm{Ne}}1}+\frac{1}{{\rm{Ne}}2}+\frac{1}{{\rm{Ne}}3}\ldots \frac{{\rm{N}}}{{\rm{Net}}-1}}$. From the ratio between molecular heterozygosity expected for F8 (He) and for F0 (H0), we calculated the remaining genetic diversity (rGD), considering the effective population size, and then we estimated the genetic diversity for F0.

Allelic richness (Ra) and inbreeding coefficient (f) were calculated using Fstat version 2.9.3.2^80^. The mean relatedness (r_m_) between individuals was estimated using Coancestry^81^. This software calculates seven different relatedness estimators, and after testing all of them, we choose the estimator based on Triade likelihood (TrioML), which showed the smallest variance among all the estimators tested^81^.

Individual heterozygosity, based on the internal relatedness index (IR)^82^ was calculated for the individuals recently transferred from Brazil to England, using GENHET^83^, in order to add a relevant molecular genetic diversity parameter to the *MSI* obtained from pedigree data.

## Supplementary information

Supplementary information.
