# Supplementary material for: Studbook and molecular analyses for the endangered black-lion-tamarin; an integrative approach for assessing genetic diversity and driving management in captivity
Source: Sci Rep. 2020 Apr 22;10:6781. doi: 10.1038/s41598-020-63542-2 (PMC7176676; doi:10.1038/s41598-020-63542-2)
Supplement: Supplementary file 1 — Supplementary information. [file 41598_2020_63542_MOESM1_ESM.docx]

**Studbook and molecular analyses for the endangered black-lion-tamarin; an integrative approach for assessing genetic diversity and driving management in captivity**

# Paola Andrea Ayala-Burbano^1^*, Pedro Manoel Galetti Jr^1^, Dominic Wormell^2^, Alcides Pissinatti^3^, Mara Cristina Marques^4^, Patrícia Domingues de Freitas^1*^

# **SUPPLEMENTARY INFORMATION**

## **Population dynamics**

The complete pedigree of BLT from 1973 to 2018 consists of 517 animals and nine generations (Fig. S1). The proportional change in population size (ƛ) from a year to the next, based on the life table calculations, and the instantaneous rate of population change (*r*), measured for males and females, was ƛ =1.016 and *r* = 0.016 for the Whole Captive Population (WCP); ƛ = 1.158 and *r* = 0.072 for the Current Captive Population (CCP); and ƛ = 1.075 and *r* = 0.147 for the Brazilian Captive Population (BCP). The total life expectancy observed for BCP (7.3 years) and CCP (7.4 years) was similar, although differences between males and females within BCP (10.1 years and 4.4 years, respectively) and within CCP (9.9 years and 5 years, respectively) were found. It was clearly observed that the captive population of BLT is aging and showing a male bias, with a lower number of females (Fig. S2A). The current captive population shows a non-stable age structure, since a female deficit was observed in the 0-3 years age class (Fig. S2B).

Demographically, the whole captive population of BLT began to decline, especially in the number of females from the year 2000 (Fig. S3). Nowadays, the age structure of the current population is relatively old, since many individuals are above 7 years old and there is no male-female balance. Thus, the structure of the captive population of BLT is typical for a slowly growing population (λ>1).

The mortality (Qx) and fertility (Mx) analyses for WCP showed a clear difference between sexes when ages were considered, gradually increasing throughout life in females (Figs. S4A and S4B). Despite such differences, the life expectancy was similar in both males and females (~7.5 years) in CCP. Annual mortality rates by age-class showed high infant mortality and similar fluctuations in both sexes up to the age 13 years (Figure S4A). In general we observed, in a way that only 50% of males and 58% of females survived to the age of first reproduction (18 months approximately) (Fig. S4C).

We observed an increase in mortality rates in some age classes for both females and males, when we considered the year 2014 (BCP) and 2018 (CCP) (Fig. S5). When we compared this years, the mortality rates showed significant differences between the sexes, according to the Mann-Whitney test (Female, U=106.5, *P=0.00*: Males, U=118, *P=0.00*).

Age-specific fertility rates showed an onset of fertility at age of 1 year for females and 2 years for males. We also observed a differential relation between reproductive peaks, considering males and females, in which fertility increases at 3 and 2 years old for the males and females, respectively (Fig. S4B). The reproduction for BLT has preferably been occurring in captivity from September to October, with the major birth peak occurring in October, and smaller peaks in May, June and July (Fig. S6). Litters with one, two or three infants were commonly observed, with a larger number of birth twins (162) than single (88) and triplets (21); and higher mortality rates for triplets (54.5%) and twins (40.7%).

# **Technical procedures for amplification and genotyping of the STR loci**

We used 15 microsatellite loci previously described for *Leontopithecus* species, from which eight were for *Leontopithecus chrysopygus,* three for *Leontopithecus rosalia,* and four for *Leontopithecus chrysomelas* (Table S3). Polymerase Chain Reactions (PCRs) were first performed using the annealing temperatures (Ta) described initially for each species, in order to test the amplification patterns and determine the expected fragment sizes for each locus. Then, annealing temperatures were adjusted for improving the amplification patterns of some loci (Table S3).

PCRs were performed in 10 µl of reaction volume, containing template 1ul of DNA (50 ng), and forward primer (0.12 pmol), reverse primer (0.46 pmol), M13 primer (0.46 pmol), MgCl_2_ (0.63mM), BSA (0.25mg/ml), and 1X GoTaq Master Mix (Promega, Madison, WI). We used an Eppendorf Mastercycler Gradient® Thermal Cycler (Eppendorf AG, Hamburg, Germany) equipment under the following programming: 5 minutes at 94 °, followed for 35 cycles of 30 s at 94 ° C, 45 s at the primer-specific annealing temperature, 45 s at 72 °C. Finally, 10 cycles of 30 seconds at 94 °C, 45 s at 53 °C (annealing temperature of M13 tail), and 45 s at 72 °C. Each locus was standardized with a specific fluorophore. M13 primers were labeled with FAM, PET, NED or VIC, following the methodology proposed by Schuelke (2000) (Table S3).

Amplification patterns were confirmed by electrophoresis using 2% agarose gels, under constant voltage (100V for 35 min). After obtaining amplicons into the expected sizes and without spurious bands, PCR-amplified products were purified using the PEG (Polyethylene glycol 20%) protocol, according to Lis & Schleif (1975); and then sequenced in an automatic sequencer ABI3730XL (Applied Biosystems, Foster City, CA, USA), aiming to confirm the microsatellite motifs for the heterologous loci. The obtained sequences were aligned and compared to the reference microsatellite sequences available in Genbank (<https://www.ncbi.nlm.nih.gov/genbank/>), using BLASTn (<https://blast.ncbi.nlm.nih.gov/Blast.cgi>). After confirming the motifs, random PCRs were performed, and the labeled products were genotyped.

***References***

Schuelke, M. An economic method for the fluorescent labeling of PCR fragments. *Nat. Biotechnol.* **18**, 233–234 (2000).

Lis, J. T. & Schleif, R. Size fractionation of double-stranded DNA by precipitation with polyethylene glycol. *Nucleic Acids Res.* **2**, 1–7 (1975).

# ***Supplementary Figures***

**Supplementary Figure S1.**


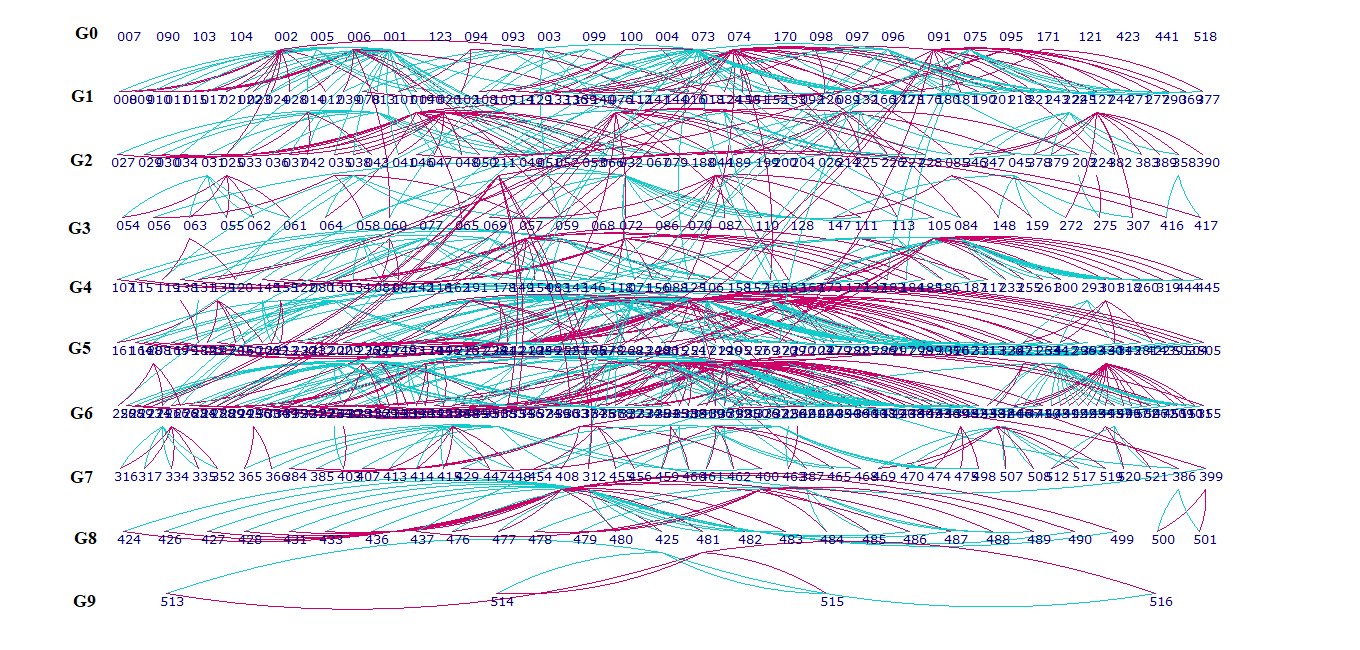


**Supplementary Figure S1.** Pedigree graphic of the whole captive population of the black-lion-tamarin according to data from Studbook. G0 represents the wild founders. G1-G9 represent the descendants. Pink lines indicate females. Blue lines indicate males.

**Supplementary Figure S2.**
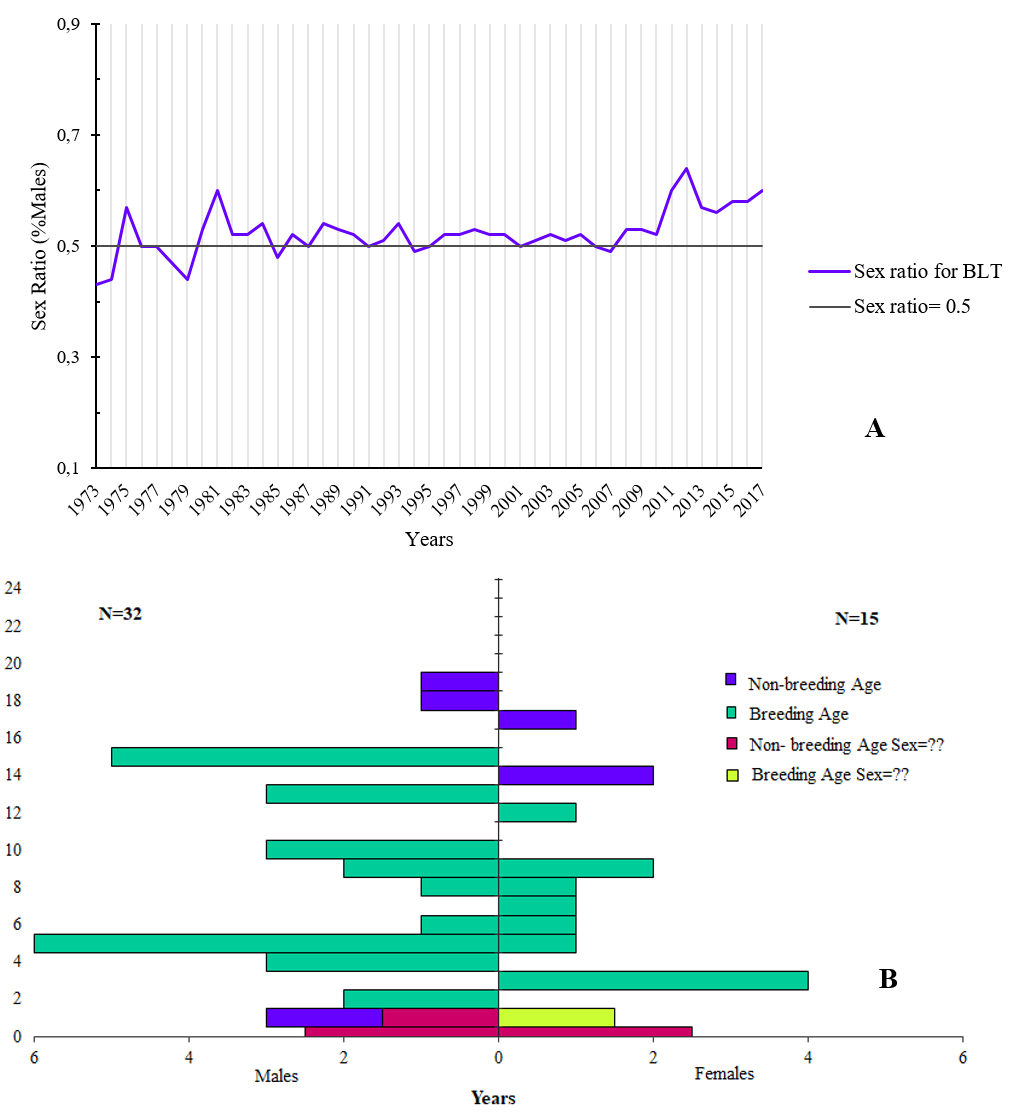


**Supplementary Figure S2.** Representation of population dynamics of black-lion- tamarin in captivity. (A) Sex ratio across years showing a male bias. (B) Age pyramid for the Current Captive Population (CCP) at the end of 2018. Number of males and females (N).

**Supplementary Figure S3.**

**Supplementary Figure S3.** Population Size Fluctuation: number of black-lion-tamarins in captivity from 1973 to 2017.

**Supplementary Figure S4.**


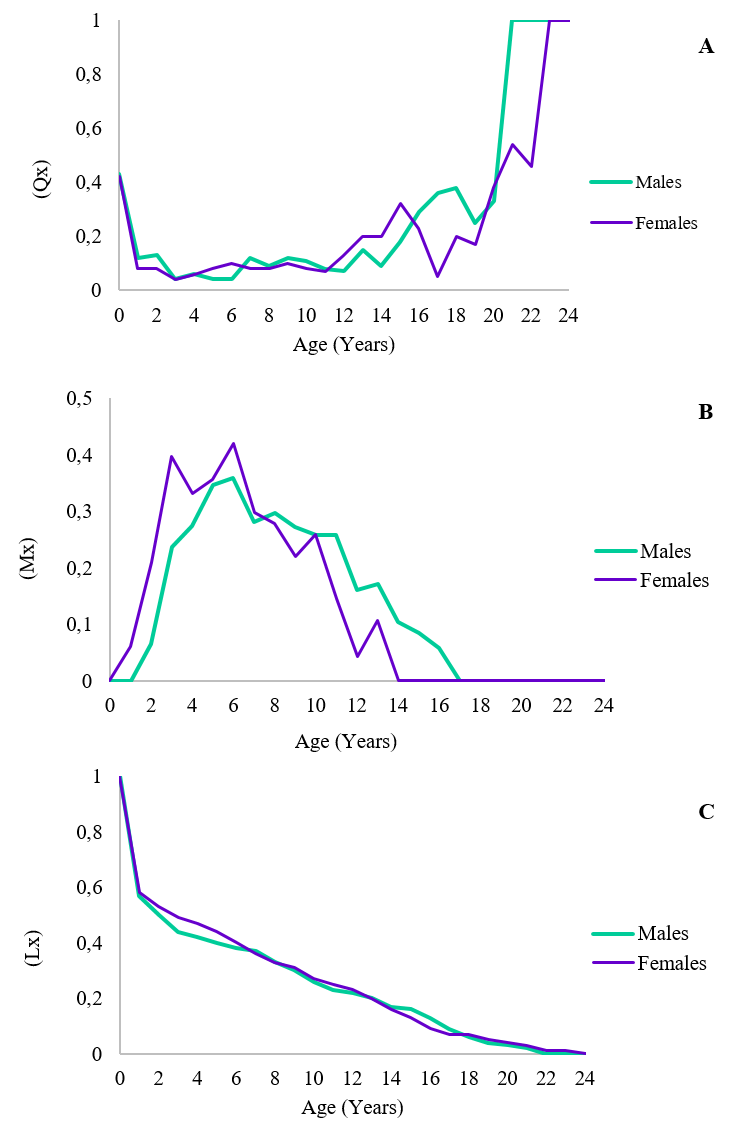


**Supplementary Figure S4**. Representation of population dynamics for the whole captive population of black-lion-tamarins. (A) Mortality rate (Qx): proportion of died individuals within an age group (B). Fecundity (Mx): the average number of same-sex young born to individuals in that age class. (C) Survivorship (Lx): proportion of individuals that survive from birth to the beginning of a given age class.

**Supplementary Figure S5.**

**Supplementary Figure S5.** Box plot for the male and female black-lion-tamarins in Brazilian Captive Population (BCP) and Current Captive Population (CCP). Note the increase in the mortality rate between 2014 and 2018 for both males and females.

**Supplementary Figure S6.**

**Supplementary Figure S6.** Histogram of number of births (N) per month for the Whole Captive Population of black-lion-tamarins.


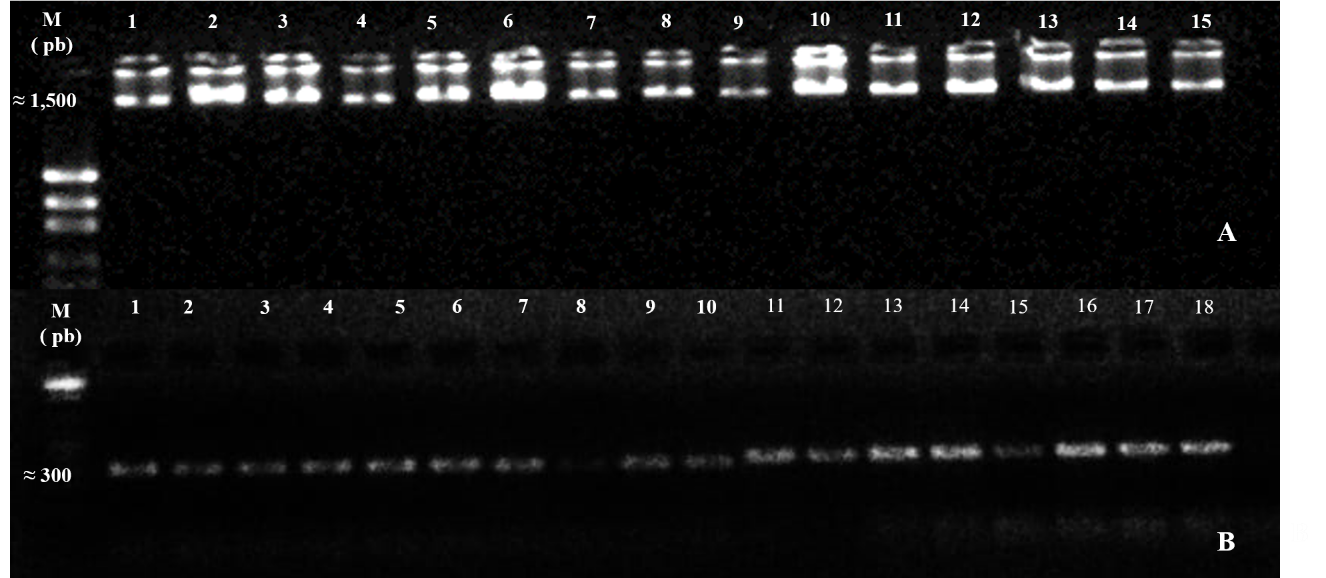
**Supplementary Figure S7.**

**Supplementary Figure S7.** (A) Agarose gel (1%) evidencing DNA profiles from blood samples of black-lion-tamarins. (B) Agarose gel (2%) showing amplicon profiles obtained for two loci, Leon 30c73 (samples 1-10) and Leon 11c72 (samples 11-18), amplified in different samples of black-lion-tamarins. M: molecular weight marker (Low DNA Mass Ladder above and 1Kb plus below)

**Supplementary Figure S8.**

**
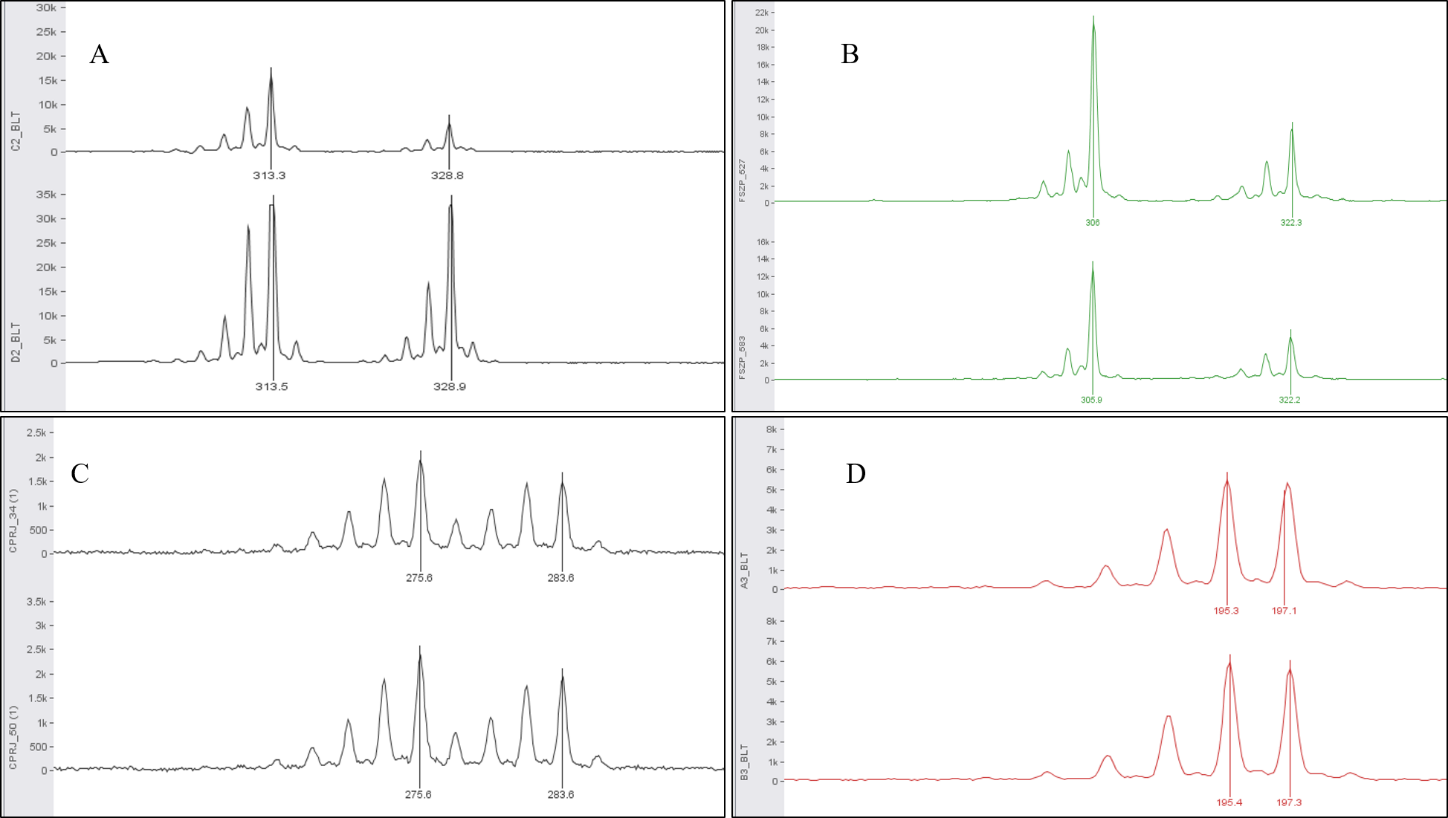
**

**Supplementary Figure S8.** Electropherograms (EPGs) showing heterozygous patterns evidencing two alleles amplified in DNA samples of black-lion-tamarins for the loci: (A) Leon 3c20 labeled with NED; (B) 11c72 labeled with VIC; (C) Leon30c72 labeled with NED; and (D) Lchu06 labeled with PET.

***Supplementary Tables***

**Supplementary Table S1**.

| Studbook number | Sex | Birth date | Death date | Zoo in 2014 | Transfer to | Population 2014 | Population 2018 |
| --- | --- | --- | --- | --- | --- | --- | --- |
| 391 | M | 12/01/2002 |  | PESC |  | BCP-2014 | CCP-2018 |
| 500 | M | 09/10/2010 | 06/03/2013 | PESC |  | BCP-2014 |  |
| 352 | F | 30/09/1999 |  | PESC |  | BCP-2014 | CCP-2018 |
| 501 | M | 11/03/2013 |  | PESC |  | BCP-2014 | CCP-2018 |
| 386 | M | 31/10/2001 |  | PESC | FPZSP | BCP-2014 | CCP-2018 |
| 399 | F | 19/09/2002 |  | PESC | FPZSP | BCP-2014 | CCP-2018 |
| 385 | M | 31/10/2001 |  | FPZSP | BH | BCP-2014 | CCP-2018 |
| 421 | F | 04/10/2004 | 02/12/2015 | FPZSP |  | BCP-2014 |  |
| 422 | F | 04/10/2004 |  | FPZSP |  | BCP-2014 | CCP-2018 |
| 458 | F | 09/01/2008 |  | FPZSP |  | BCP-2014 | CCP-2018 |
| 457 | M | 09/01/2008 |  | FPZSP |  | BCP-2014 | CCP-2018 |
| 392 | M | 12/01/2002 |  | FPZSP |  | BCP-2014 | CCP-2018 |
| 263 | M | 30/08/1995 | 28/08/2016 | FPZSP |  | BCP-2014 |  |
| 464 | M | Wild |  | FPZSP |  | BCP-2014 | CCP-2018 |
| 432 | F | 11/04/2007 |  | FPZSP |  | BCP-2014 | CCP-2018 |
| 469 | M | 13/08/2011 | 19/09/2012 | FPZSP |  | BCP-2014 |  |
| 470 | M | 13/08/2011 |  | FPZSP |  | BCP-2014 | CCP-2018 |
| 412 | M | 17/092003 |  | FPZSP |  | BCP-2014 | CCP-2018 |
| 467 | F | 17/09/2010 |  | FPZSP |  | BCP-2014 | CCP-2018 |
| 473 | M | 31/01/2012 |  | FPZSP |  | BCP-2014 | CCP-2018 |
| 430 | F | Wild |  | FPZSP |  | BCP-2014 | CCP-2018 |
| 471 | M | 20/09/2011 |  | FPZSP | Jersey | BCP-2014 | CCP-2018 |
| 472 | M | 30/01/2012 |  | FPZSP | Jersey | BCP-2014 | CCP-2018 |
| 491 | M | 10/09/2012 |  | FPZSP |  |  | CCP-2018 |
| 492 | M | 10/09/2012 |  | FPZSP |  |  | CCP-2018 |
| 495 | F | 13/08/2013 |  | FPZSP |  |  | CCP-2018 |
| 496 | F | 26/12/2013 |  | FPZSP |  |  | CCP-2018 |
| TE64 | M | 03/03/2015 |  | FPZSP |  |  | CCP-2018 |
| TE65 | U | 31/08/2015 |  | FPZSP |  |  | CCP-2018 |
| TE66 | U | 31/08/2015 |  | FPZSP |  |  | CCP-2018 |
| TE67 | U | 11/11/2015 |  | FPZSP |  |  | CCP-2018 |
| TE72 | M | 05/02/2016 |  | FPZSP |  |  | CCP-2018 |
| TE73 | M | 05/11/2014 |  | FPZSP |  |  | CCP-2018 |
| TE74 | U | 23/09/2016 |  | FPZSP |  |  | CCP-2018 |
| TE75 | U | 27/10/2016 |  | FPZSP |  |  | CCP-2018 |
| TE76 | U | 27/10/2016 |  | FPZSP |  |  | CCP-2018 |
| 502 | F | 26/12/2013 |  | FPZSP | BH |  | CCP-2018 |
| 312 | M | 11/10/1997 |  | CPRJ |  | BCP-2014 | CCP-2018 |
| 408 | F | 18/02/2003 |  | CPRJ |  | BCP-2014 | CCP-2018 |
| 486 | M | 02/10/2011 |  | CPRJ |  | BCP-2014 | CCP-2018 |
| 487 | M | 02/10/2011 |  | CPRJ | Jersey | BCP-2014 | CCP-2018 |
| 427 | M | 13/02/2007 |  | CPRJ |  | BCP-2014 | CCP-2018 |
| 366 | F | 08/11/2000 | 13/06/2016 | CPRJ |  | BCP-2014 |  |
| 384 | M | 31/10/2001 | 09/04/2015 | CPRJ |  | BCP-2014 |  |
| 436 | F | 29/09/2008 |  | CPRJ | Jersey | BCP-2014 | CCP-2018 |
| 387 | M | 31/10/2001 |  | CPRJ |  | BCP-2014 | CCP-2018 |
| 400 | F | 19/09/2002 | 16/08/2015 | CPRJ |  | BCP-2014 |  |
| 488 | M | 02/10/2011 |  | CPRJ |  | BCP-2014 | CCP-2018 |
| 437 | M | 27/10/2008 |  | CPRJ |  | BCP-2014 | CCP-2018 |
| 481 | F | 01/01/2010 |  | CPRJ |  | BCP-2014 | CCP-2018 |
| 431 | M | 26/10/2007 |  | CPRJ |  | BCP-2014 | CCP-2018 |
| 424 | M | 14/12/2005 | 22/07/2014 | CPRJ |  | BCP-2014 |  |
| 425 | M | 27/12/2006 |  | CPRJ |  | BCP-2014 | CCP-2018 |
| 428 | M | 13/02/2007 |  | CPRJ |  | BCP-2014 | CCP-2018 |
| TE68 | M | 06/01/2016 |  | CPRJ |  |  | CCP-2018 |
| TE69 | M | 06/01/2016 |  | CPRJ |  |  | CCP-2018 |
| TE70 | U | 05/12/2016 |  | CPRJ |  |  | CCP-2018 |
| TE71 | U | 05/12/2016 |  | CPRJ |  |  | CCP-2018 |
| 410 | M | 06/08/2003 |  | Jersey |  |  | CCP-2018 |
| 419 | M | 10/02/2004 |  | Jersey |  |  | CCP-2018 |
| 468 | M | 22/03/2011 |  | Jersey |  |  | CCP-2018 |
| 497 | F | 23/12/2013 |  | Jersey |  |  | CCP-2018 |
| 333 | M | 15/11/1998 |  | Mag |  |  | CCP-2018 |

**Supplementary Table S1**. Summary for the captive populations of the black-lion-tamarins in 2014 and 2018.

*São Carlos Ecological Park (PESC, SP); Zoological Park Foundation of São Paulo State (FPZSP, SP); Primatology Center of Rio de Janeiro (CPRJ, RJ); Jersey Zoo, Belo Horizonte Zoo (BH); Magdeburg Zoo (Mag).

**Supplementary Table S2.**

| Year | 1974 | 1975 | 1976 | 1977 | 1978 | 1979 | 1980 | 1981 | 1982 |
| --- | --- | --- | --- | --- | --- | --- | --- | --- | --- |
| Inbreeding | 0 | 0 | 0 | 0 | 0 | 0 | 0 | 0 | 0 |
| Year | **1983** | **1984** | **1985** | **1986** | **1987** | **1988** | **1989** | **1990** | **1991** |
| Inbreeding | 0 | 0,0119 | 0,0208 | 0,026 | 0,029 | 0,0337 | 0,0313 | 0,037 | 0,0318 |
| Year | **1992** | **1993** | **1994** | **1995** | **1996** | **1997** | **1998** | **1999** | **2000** |
| Inbreeding | 0,0229 | 0,0204 | 0,0185 | 0,0199 | 0,0212 | 0,0223 | 0,0268 | 0,0397 | 0,0408 |
| Year | **2001** | **2002** | **2003** | **2004** | **2005** | **2006** | **2007** | **2008** | **2009** |
| Inbreeding | 0,0414 | 0,0428 | 0,0483 | 0,0486 | 0,0519 | 0,0541 | 0,0574 | 0,0656 | 0,0702 |
| Year | **2010** | **2011** | **2012** | **2013** | **2014** | **2015** | **2016** | **2017** | **2018** |
| Inbreeding | 0,0788 | 0,086 | 0,0952 | 0,095 | 0,0916 | 0,0887 | 0,0874 | 0,107 | 0,107 |

**Supplementary Table S2.** Inbreeding coefficient values over the years for the whole captive population of black-lion-tamarins.

**Supplementary Table S3.**

| Locus | Primer Sequence (5`-3`) | Repetitive unit | Annealing Temperature (°C) | PCR product size range | ABI Label | GenBank Acess | Source |
| --- | --- | --- | --- | --- | --- | --- | --- |
| *Leon2^a^ | F: CTGCTTCTTGTTCCACTTCTTCTC  R: GTTTGGGTGGTTGCCAAG | (CA)_18_(CG)(CA)_3_ | 56 | 219-223 | FAM | AY706915 | Perez-Sweeney et al. 2005 |
| *Leon15c85 ^a^ | F: CTGCTTCTTGTTCCACTTCTTCTC  R: GTTTGGGTGGTTGCCAAG | (GA)_17_ | 60 | 284-296 | FAM | AY706920 | Perez-Sweeney et al. 2005 |
| *Leon3c20 ^a^ | F: CTGTATGTGATCGCTTTTACCTG  R: AAGGCAATCTAACTAATCAACACTC | (GT)_22_ | 60 | 312-318 | NED | AY706916 | Perez-Sweeney et al. 2005 |
| *Leon21c75 ^a^ | F: CAGTTGAGGGAACAGGAATTA  R: CACTGCACTGACAGAGCAAG | (GT)_19_(NA)1(GT)_5_ | 60 | 294-298 | FAM | AY706922 | Perez-Sweeney et al. 2005 |
| *Leon30c73 ^a^ | F: GGACCTGATTGAAGCAGTC  R: TTCCCTGAGAATCTAATGGAG | (TC)_25_(AA)(TC)(TG)_16_ | 60 | 274-284 | NED | AY706927 | Perez-Sweeney et al. 2005 |
| *Leon31c97 ^a^ | F: TGGTCCAGAGAAATGATGTC  R: GTAATTCCTTGGATTTATGCC | (GA)_2_(CA)_2_(GA)_19_(TT)(GA)(CA)_4_ | 55 | 328-340 | PET | AY706928 | Perez-Sweeney et al. 2005 |
| *Leon11c72 ^a^ | F: AGGATTACAGGTGCCCAC  R: TTGCATATTGTGTTCAACTTC | (GT)_21_ | 60 | 307-323 | VIC | AY706921 | Perez-Sweeney et al. 2005 |
| *Leon35c42 ^a^ | F: GTGGAAAGGTTTCAGAATATC  R: TGCAGTTGTCCACACTTTA | (CT)_16_(CA)_9_(T)(AC)_3_ | 58/60 | 219-223 | FAM | AY706929 | Perez-Sweeney et al. 2005 |
| Leon26c10 ^a^ | F: TTCATCTCAATGACACGAAAC  R: CATCGAGTGTCCTGCTGT | (TG)_17_(AG)_15_(GT)_3_(CT)(GT) | 58 | 266 | VIC | AY706924 | Perez-Sweeney et al. 2005 |
| Leon27c13 ^a^ | F: AAGCGCAGATTTATTGATAGG  R: TGCAGGTAAATGATGGTAATG | (CA)_11_ | 60 | 213 | PET | AY706925 | Perez-Sweeney et al. 2005 |
| *Lchu01 ^b^ | F: GCTCAGGTGTTATTTATGTCCAAA  R: GTTTCTTGCAACTATCTTGCATGTTCTGC | (TTTA)_8_ | 58 | 213-225 | FAM | DQ979343 | Galbusera and Gillemot 2007 |
| Lchu02 ^b^ | F: AGATTCTGCCTCAAGAAATTCAGT  R: GTTTCTTTCTAGATCCAGGTCGGCAAT | (A)_3_ T(AGAA)_2_ | 60 | 314 | FAM | DQ979344 | Galbusera and Gillemot 2007 |
| Lchu03 ^b^ | F: AAGGCATGATGTATCTTGTTCTCA  R:GTTTCTTATCTTTCTGTATGTGTCTCCCTGTCT | (GATA)_13_ | 58 | 336 | VIC | DQ979345 | Galbusera and Gillemot 2007 |
| Lchu04 ^b^ | F: TGACCAAAGAAAATGCAAAA  R: GTTTCTTGCACAGGGTATTTAGCAGGA | (AGAT)_14_ | 58 | 396-400 | VIC | DQ979346 | Galbusera and Gillemot 2007 |
| Lchu05 ^b^ | F: TGATGCTAAAACAGAAGCATTT  R: GTTTCTTGTCCTGATGTTCACAAAACCT | (GAAG)11 | 58 | 256 | VIC | DQ979348 | Galbusera and Gillemot 2007 |
| *Lchu06 ^b^ | F: GCCTTAATTAGCACCAGAACC  R: GTTTCTTACCACTCCAAGCCTTCAGTA | (CA)8 | 55 | 193-195 | PET | DQ979349 | Galbusera and Gillemot 2007 |
| *Lchu07 ^b^ | F: TCTCATTTCTTCTCATGGACTC  R: GTTTCTTCTTGACTCACAGCATGACCT | (TG)_16_ | 55 | 342-348 | FAM | DQ979350 | Galbusera and Gillemot 2007 |
| *Lchu08 ^b^ | F: CACGGCAATGTGGGAATAA  R:GTTTCTTTTCAGTAGTTGGGACTGGGATAA | (TG)_23_ | 58 | 224-236 | VIC | EF583690 | Galbusera and Gillemot 2007 |
| Lchu09 ^b^ | F: TTCATTGTAGCATTGTTGGTCAT  R: GTTTCTTTTGCCTCCTCATAGTTCCTCAT | (CA)_19_ | 58 | 418-422 | FAM | EF583691 | Galbusera and Gillemot 2007 |
| *Lr. P2BH6 ^c^ | F: TCTGTTTGAATCCCCAGTCC  R: GCAGTCCCTCAAGGTTTTCT | (CA)_19_ | 58 | 132-136 | PET | AF320577 | Grativol et al. 2001 |
| *Lr. P5BE6 ^c^ | F: TGTGCATGCTTGCCTGTGTC  R: ATCTCACTGGACCCACCT | (CA)_27_ | 58 | 120-130 | PET | AF320580 | Grativol et al. 2001 |
| *Lr. P3AF1 ^c^ | F: CCATCCTGGCCAACATAGGT  R: GTAGCTGGGATTACAGGCAC | (CA)_23_ | 62 | 126-130 | FAM | AF320581 | Grativol et al. 2001 |

Specific primers developed for ^a^ *Leontopithecus chrysopygus*; and heterologous primers developed for ^b^ *Leontopithecus chrysomelas*, and **^c^** *Leontopithecus rosalia*.

*Polymorphic loci selected to be used for the subsequent analyses of genetic diversity in the black-lion-tamarins from captivity.

**Supplementary Table S3.** Summary information on the homologous and heterologous microsatellite loci validated for the black-lion-tamarin.
